# The relative spinal cord float back distance might be associated with C5 palsy after posterior cervical decompression

**DOI:** 10.3389/fsurg.2026.1816262

**Published:** 2026-08-03

**Authors:** Ding Yue, Yue Huang, Yuanyuan Lu, Lingyun Yang, Yucheng Shen, Hu Qin, Xiaojie Mei, Tianyu Jiao, Yue Gu, Tao Wu

**Affiliations:** 1 Department of Spinal Surgery, The Second Affiliated Hospital of Nanjing Medical University, Nanjing, China; 2 Department of Orthopedics, Binhai County People's Hospital, Yancheng, China

**Keywords:** C5 palsy, cervical spondylotic myelopathy, laminectomy, laminoplasty, the distance of relative spinal cord float back

## Abstract

**Objective:**

To investigate the independent risk factors for C5 palsy (C5P) after cervical posterior decompression.

**Methods:**

Between 2020 and 2023, 97 patients with multisegmented cervical spondylotic myelopathy (CSM) who underwent posterior cervical decompression surgery at our hospital were retrospectively reviewed. Clinical features and radiological data of patients with and without C5P were analyzed. The clinical features of the patients who underwent two different surgeries were analyzed. Then the parsimonious multivariable logistic regression was conducted. Receiver operating characteristic (ROC) curve analysis was performed to evaluate the diagnostic performance of the relative spinal cord float back distance (RSD) and intervertebral foramen diameter (IFD). Kaplan–Meier analysis was used to compare C5P recovery time between different surgical approaches and RSD subgroups.

**Results:**

Patients in C5P group had higher RSD and narrower IFD than non-palsy group. Patients in laminoplasty group had lower postoperative VAS scores for axial pain and lower incidence of C5P. Parsimonious multivariate binary logistic regression analysis showed that RSD was associated with increased odds of C5P. ROC analysis further demonstrated that an RSD > 6.91 mm predicted postoperative C5P with an accuracy of 85.00%. Kaplan–Meier analysis revealed that, in the laminoplasty group, patients presenting with C5P and RSD < 6.91 mm experienced significantly shorter recovery times compared to the laminectomy group.

**Conclusion:**

RSD might be associated with increased odds of C5P, and the IFD might be associated with decreased odds of C5P. These results indicated that any potential factor that may induce traction on the C5 nerve root could contribute to the development of C5P. Laminoplasty might have lower odds of C5P compared with laminectomy.

## 1 Introduction

Cervical posterior decompression surgery is widely used to treat multisegmented CSM. Although clinical practice has confirmed that this surgical approach could achieve great therapeutic effects, it is often accompanied by C5P after surgery. C5P is characterized by a decrease in the strength of the deltoid muscle, difficulty in arm abduction, biceps brachii paralysis, and sensory abnormalities (pain or numbness) in the area dominated by the C5 nerve root, which are usually bilateral in the upper limbs, but there are no signs of lower limb muscle strength or sensory changes (1). There have been many studies on the risk factors for C5P; However, the pathogenesis of C5P remains unclear. Among these risk factors, spinal cord float back has been confirmed by many researchers as an important risk factor for C5P (2–5). Some studies have shown that change in cervical curvature (ΔCCD) (6) is also an independent risk factor for C5P. ΔCCD might induce a bowstring effect (7–9), subsequently increasing posterior spinal cord float back. This mechanism may be considered an indirect factor contributing to spinal cord float back. However, there are currently no radiological parameters that could simultaneously account for the impact of spinal cord float back and cervical curvature changes on C5P. Therefore, this study innovatively introduces a new radiographic parameter called relative spinal cord float back distance by combining ΔCCD and spinal cord float back distance (SD). We collected clinical features and radiological data from patients with CSM who underwent cervical posterior decompression surgery at our hospital and conducted a retrospective study to explore the independent risk factors for postoperative C5P.

## 2 Material and methods

### 2.1 General information

A total of 120 patients with multisegmented CSM who underwent posterior cervical decompression surgery in our department between September 2020 and September 2023 were included.

Inclusion criteria: ① Patients who underwent posterior cervical laminoplasty or total laminectomy. ② Complete preoperative and postoperative imaging and clinical data. ③ The surgical range must include the C4/5 intervertebral space. ④ The follow-up time is not less than 6 months.

Exclusion criteria: ① Patients with a history of cervical surgery. ② Patients with cervical tumors or infections. ③ Patients with cervical deformities (including those with unstable cervical vertebrae). ④ Patients with intellectual disabilities or those unable to accurately describe their symptoms. ⑤ Patients with preoperative frozen shoulders or rotator cuff injury. ⑥ Patients with ossification of the posterior longitudinal ligament (OPLL).

Based on the selection criteria, 97 patients were included and retrospectively assessed. During the follow-up period, 15 patients (15.46%) developed postoperative C5P, among whom 2 patients still had symptoms even after 6 months of follow-up. C5P is defined as new postoperative deltoid and/or biceps weakness, with or without sensory disturbance.

### 2.2 Surgical approach

To minimize performance bias, all surgical procedures were performed by the same senior surgeon using the same type of imported instrumentation (Medtronic) between September 2020 and September 2023. The choice of surgical procedure was determined based on the patient's preoperative cervical alignment, stability, and pathological severity. Generally, cervical posterior open-door laminoplasty was indicated for patients with preserved cervical lordosis and absence of segmental instability. Conversely, total laminectomy with screw fixation was reserved for patients exhibiting local kyphosis or segmental instability.

#### 2.2.1 Cervical posterior open-door laminoplasty

After general anesthesia, the patient was placed in a prone position on a plaster bed, with the shoulders pulled backward and the neck slightly flexed. After disinfection and draping of the surgical area, a median incision was made on the posterior aspect of the neck and cut through the skin and subcutaneous tissues. The paravertebral muscles on both sides were stripped subperiosteally along the ligamentum nuchae, exposing the vertebral lamina and the lateral masses. A double-cortex cut was made at the outer edge of the vertebral lamina using a high-speed grinder and a bone nibbler as the door opening side. On the other side, a single-cortex cut was made at the outer edge of the vertebral lamina using a high-speed grinder as the hinge side. The spinous process was pushed away with a periosteal elevator, allowing one side of the vertebral lamina to open and expose the dura mater, which gradually bulged due to compression. A mini titanium plate (Medtronic) was used to fix the spinous process and lateral mass on the door opening side while maintaining the open position. The position of internal fixation was confirmed satisfactorily using C-arm fluoroscopy. Hemostasis was achieved, the wound surface was irrigated, a drainage tube was placed inside, and the incision was closed layer by layer.

#### 2.2.2 Cervical posterior total laminectomy with screw fixation

The patient’s anesthesia, positioning, and surgical exposure methods were the same as before. After exposing the vertebral lamina and lateral masses, spinous processes behind the responsible segments were removed. Lateral mass screws or pedicle screws (Medtronic) were implanted on both sides of the decompressed vertebrae in sequence and connected and fixed with connecting rods (Medtronic) of appropriate length and curvature. The double cortex of the bilateral vertebral lamella was cut along the outer edges using a high-speed grinder and a bone nibbler, and the vertebral lamina was removed to expose the dura. The compressed dura mater gradually bulged, and the spinal canal was unobstructed. The position of internal fixation was confirmed satisfactorily under C-arm fluoroscopy, the wound surface was irrigated, a drainage tube was placed inside, and the incision was closed layer-by-layer.

### 2.3 Data collection and measurement methods

The neurological assessment was based on the mJOA score for cervical myelopathy. The rate of neurological improvement 6 months after surgery was calculated as follows: [(postoperative mJOA score − preoperative mJOA score)/(17 − preoperative mJOA score) × 100%]. The Postoperative Neck Axial Pain VAS Score was filled out by all the patients at the 6th month follow-up after surgery based on visual analogue scale. The clinical relief of C5P was defined by deltoid strength returning to grade 5, and the sensory symptoms were not included in the recovery endpoint. Patients who did not recover were censored based on the last follow-up.

**Figure 1:**
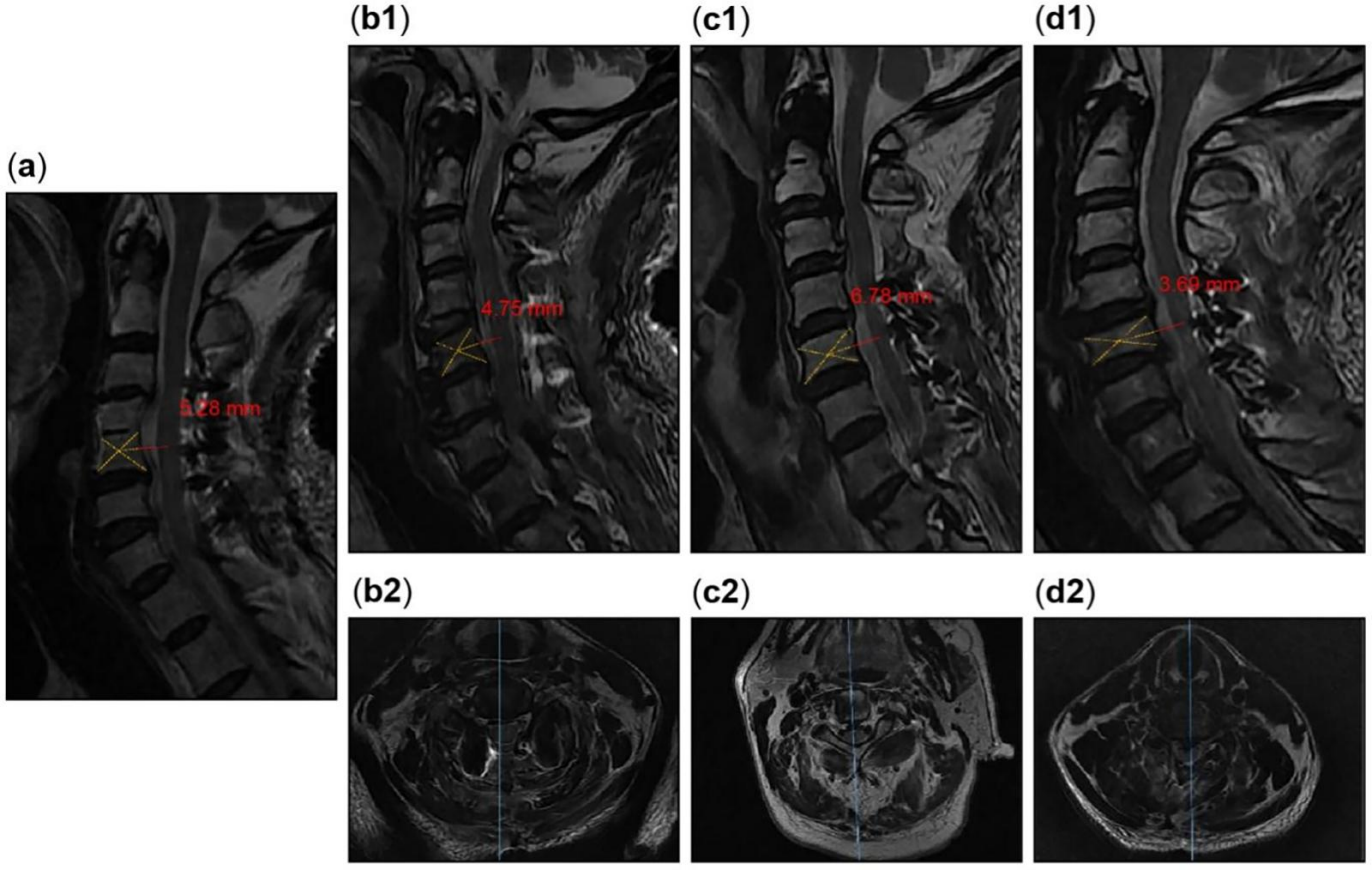
**(a)** The measurement method for the spinal cord float back distance (SD) on the mid-sagittal MRI. The reference line (yellow dotted line) connected between the center of the vertebral body and its posterior edge. This line was then extended to the center of the spinal cord (red dotted line), and the distance of this extension was defined as SD. The SD was measured at C5 level. **(b1,b2)** Patients with C5P after total laminectomy: sagittal and coronal MR images. **(c1,c2)** Patients with C5P after Cervical Posterior Laminoplasty: sagittal and coronal MR images. **(d1,d2)** Patients without C5P after Cervical Posterior Laminoplasty: sagittal and coronal MR images (This measurement technique is applicable to vertebrae with stable alignment; severe deformities were excluded to maintain reproducibility).

**Figure 2:**
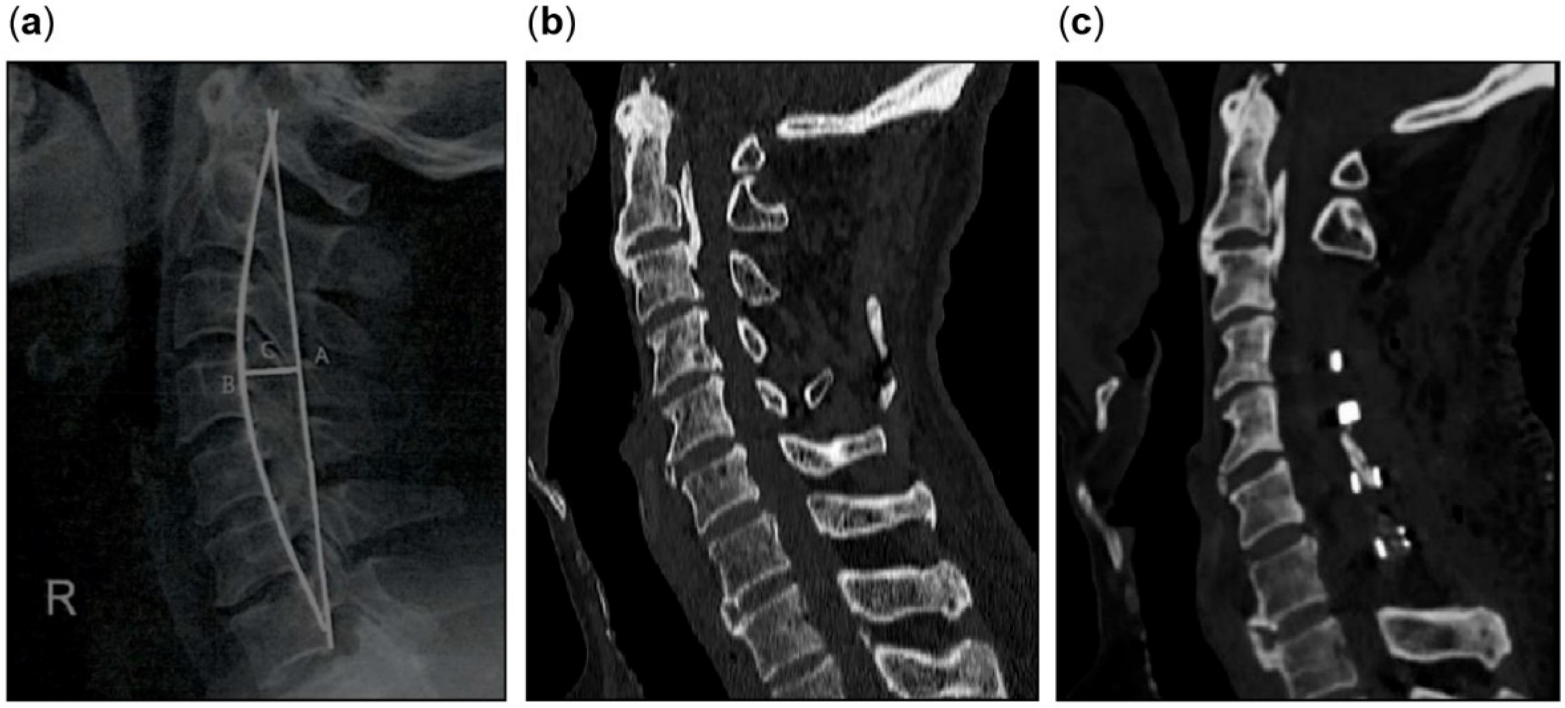
**(a)** The measurement method of cervical curvature (CCD). **(b,c)** Preoperative and postoperative cervical sagittal position of Cervical Posterior Laminoplasty. Line A was drawn from the posterosuperior odontoid tip of C2 to the posteroinferior corner of C7. Curve B followed the posterior vertebral body margins. The maximum perpendicular distance from the curve's apex to line A was measured and designated as CCD.

**Figure 3:**
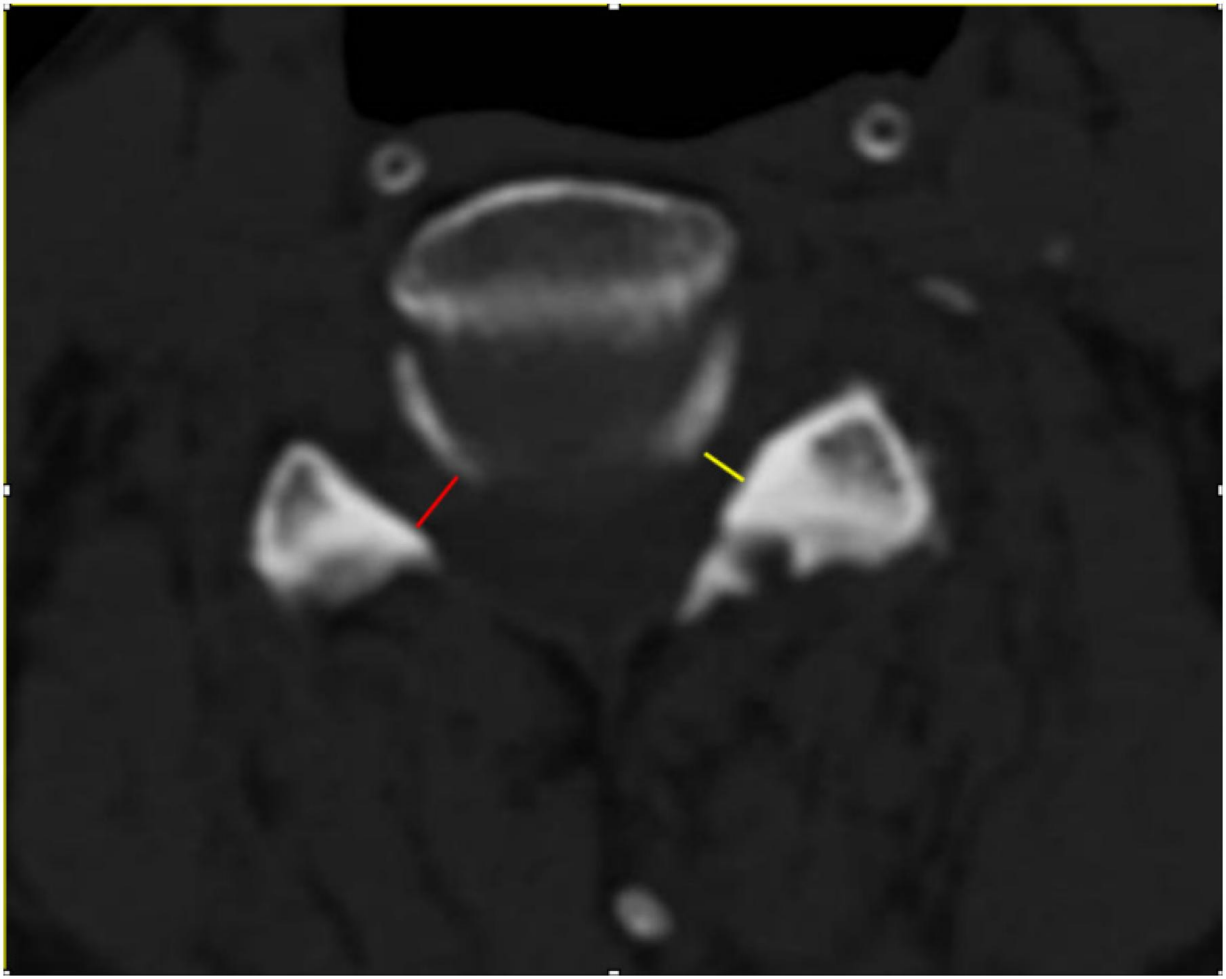
The intervertebral foramen diameter (IFD) on C4/5 level. IFD was calculated as the average of the intervertebral foramen diameters measured on both sides (the yellow line and the red line).

All radiological data were measured three times by three spinal surgeons, and the average of all data was calculated. The three spinal surgeons demonstrated high inter-observer reliability (ICC = 0.89) and intra-observer reproducibility (ICC = 0.91) for the measurement of primary radiographic parameters. Consequently, the derived parameter, RSD, maintains a high level of measurement stability and reproducibility across different observers. On mid-sagittal T2-weighted MRI studies at 1-week after surgery, the spinal cord float back distance (SD) was measured using our methods (Figure 1). The “center of the spinal cord” was defined as the geometric midpoint between the anterior and posterior margins of the cord. The distance from the extension line of the connection between the center of the vertebral body and the posterior edge of the vertebral body to the center of the spinal cord was defined as SD. To ensure measurement reliability, patients with significant rotational deformity or spondylolisthesis were excluded from this study (Exclusion Criteria #3), as these conditions would compromise the accuracy of the reference line. The BRODEN method was used to measure the cervical curvature (CCD) on plain radiographs or sagittal CT scans in this study (Figure 2). On axial CT scans, the average width of the intervertebral foramen at C5 (Figure 3) was used to determine the intervertebral foramen diameter (IFD). Finally, the relative spinal cord float back distance (RSD) was calculated as the sum of ΔCCD (post-CCD—pre-CCD) and SD.

### 2.4 Research methods

Statistical analyses were performed using SPSS version 25.0. Data are expressed as mean ± SD. The independent sample *T* test was used for comparisons between two groups, and the chi-square test was used for comparisons of categorical data. Given the small number of C5P events (*n* = 15) and to minimize the risk of model overfitting, a parsimonious multivariable logistic regression model that included only the two variables with the strongest clinical and univariable associations (RSD and IFD) was constructed. Then, the receiver operating characteristic (ROC) curve and area under the curve (AUC) were used to evaluate the value of independent risk factors in predicting the occurrence of postoperative C5P. Finally, the Kaplan–Meier plot was used to assess the impact of RSD and surgical methods on C5P prognosis during the follow-up period. The endpoint of survival was spontaneous clinical relief of C5P. Patients who did not recover were censored based on the last follow-up.

## 3 Results

### 3.1 Univariate analysis between the paralysis group and the non-paralysis group

The results of the univariate analysis between C5P and non-palsy groups were shown in Table 1. We found that patients with C5P had significantly higher RSD (*P* < 0.01) and lower IFD (*P* < 0.01) than those patients without C5P. Patients who accepted total laminectomy were more likely to experience C5P than those who accepted laminoplasty. In terms of other radiographic parameters, there was no significant difference in pre- and post-mJOA, recovery rate of mJOA, pre- and post-CCD, sex, or age (*P* > 0.05).

**Table 1:** General information of the patients.

Variables	C5 palsy (*n* = 15)	Non-palsy (*n* = 82)	*P* value
Age (years)	57.07 ± 12.18	55.46 ± 13.83	0.67
Gender, *n* (%)			0.98
Man	7 (46.7%)	38 (46.3%)	
Woman	8 (53.3%)	44 (53.7%)	
Surgical approach, *n* (%)			0.029*
Laminectomy	11 (73.3%)	35 (42.7%)	
Laminoplasty	4 (26.7%)	47 (57.3%)	
Pre-mJOA	9.20 ± 1.32	9.46 ± 1.78	0.57
Post-mJOA	13.93 ± 0.79	13.76 ± 1.36	0.51
Recovery rate of mJOA	0.58 ± 0.15	0.56 ± 0.16	0.57
Pre-CCD (mm)	2.63 ± 0.86	3.19 ± 1.22	0.09
Post-CCD (mm)	4.72 ± 2.27	3.69 ± 1.86	0.06
RSD (mm)	7.97 ± 2.44	4.29 ± 2.53	<0.01*
IFD (mm)	2.32 ± 0.52	3.11 ± 0.66	<0.01*

Values are presented as mean ± standard deviation or number (percentage). RSD, relative spinal cord float back distance; IFD, intervertebral foramen diameter.

* Indicates statistical significance (*P* < 0.05).

### 3.2 Univariate analysis between laminoplasty group and total laminectomy group

Patients in Total Laminectomy group had higher VAS scores (*P* < 0.01), post-CCD (*P* < 0.01), ΔCCD (*P* < 0.01), RSD (*P* < 0.01) and higher incidence of C5P (*P* = 0.029) than those in Laminoplasty group, as shown in Table 2. There were no significant differences in the recovery rate of mJOA (*P* > 0.05) and pre-CCD (*P* > 0.05).

**Table 2:** Analysis of different surgical approaches.

Variables	Laminectomy (*n* = 46)	Laminoplasty (*n* = 51)	*P* value
C5P	11	4	0.029*
No C5P	35	47	
VAS score	3.07 ± 1.45	1.82 ± 0.84	<0.01*
Recovery rate of mJOA	0.58 ± 0.15	0.52 ± 0.25	0.172
Pre-CCD (mm)	2.98 ± 1.27	3.22 ± 1.11	0.33
Post-CCD (mm)	5.47 ± 1.31	2.39 ± 1.09	<0.01*
ΔCCD (mm)	2.49 ± 1.57	−0.82 ± 1.56	<0.01*
RSD (mm)	6.75 ± 2.19	3.15 ± 2.21	<0.01*

* Indicating significant differences exist.

### 3.3 Parsimonious multivariate binary logistic regression analysis

The simplified model including RSD and IFD showed that both variables remained significantly associated with C5P after adjusting for each other (Table 3). The RSD (OR = 1.726 per mm; 95% CI [1.240–2.405]; *P* < 0.01) was associated with increased odds of C5P, and IFD (OR = 0.117 per mm; 95% CI [0.028–0.491]; *P* < 0.01) was associated with decreased odds of C5P.

**Table 3:** Logistic regression analysis.

Factors	OR	95% CI	*P* value
RSD (mm)	1.726	1.240–2.405	0.001*
IFD (mm)	0.117	0.028–0.491	0.003*

* Indicating significant differences exist.

### 3.4 ROC curve analysis

**Figure 4:**
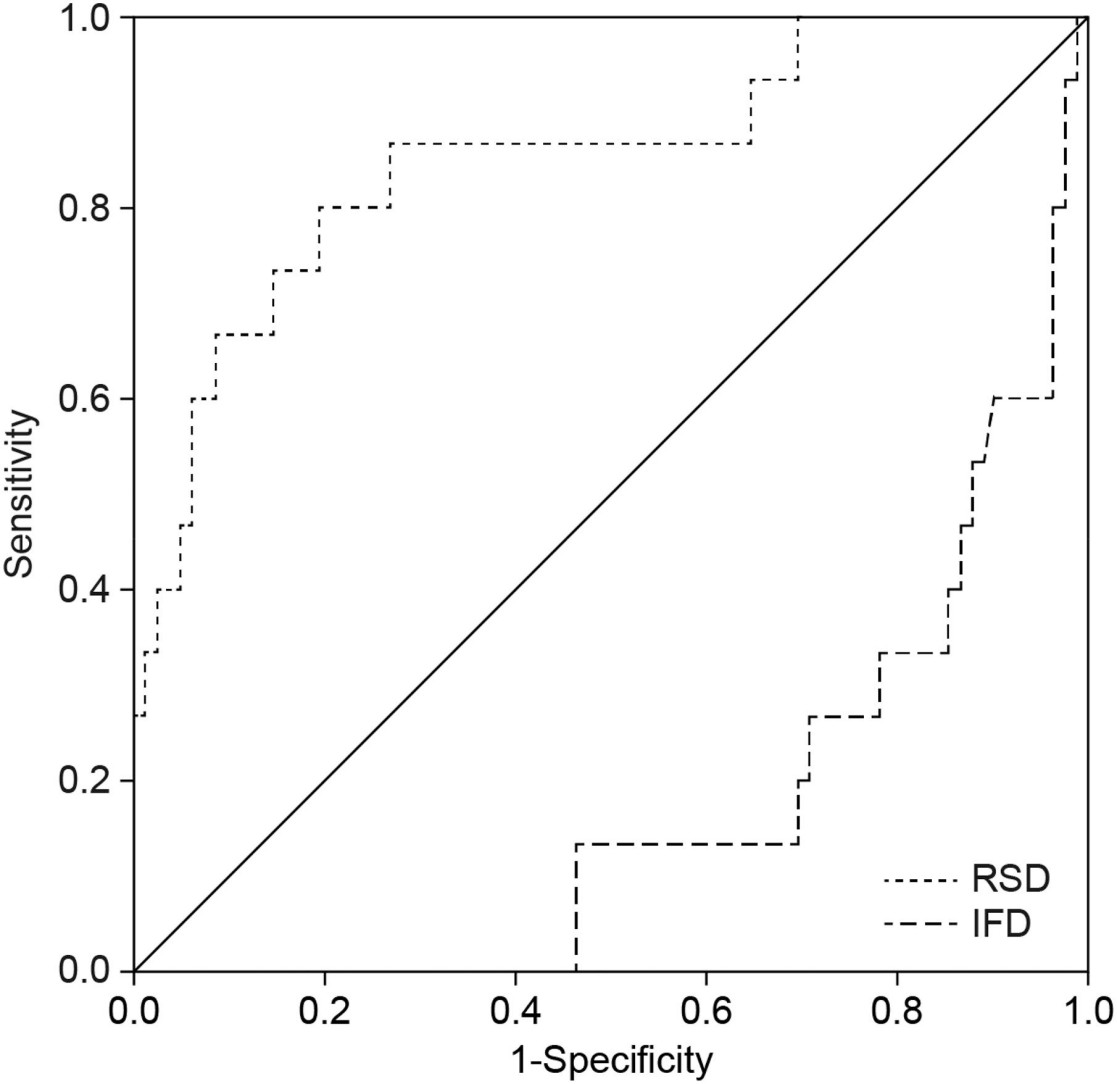
ROC curve for predicting C5P based on the RSD and IFD. The ROC curve demonstrated the predictive accuracy of RSD for C5P, with a sensitivity of 80.0%, specificity of 80.5%, and an AUC of 0.850 and the predictive accuracy of IFD for C5P, with a sensitivity of 69.5%, specificity of 86.7%, and an AUC of 0.829.

ROC curve analysis demonstrated that the RSD > 6.91 mm and IFD < 2.82 mm were significantly associated with C5P (*P* < 0.05) (Figure 4).

### 3.5 Survival analysis

**Figure 5:**
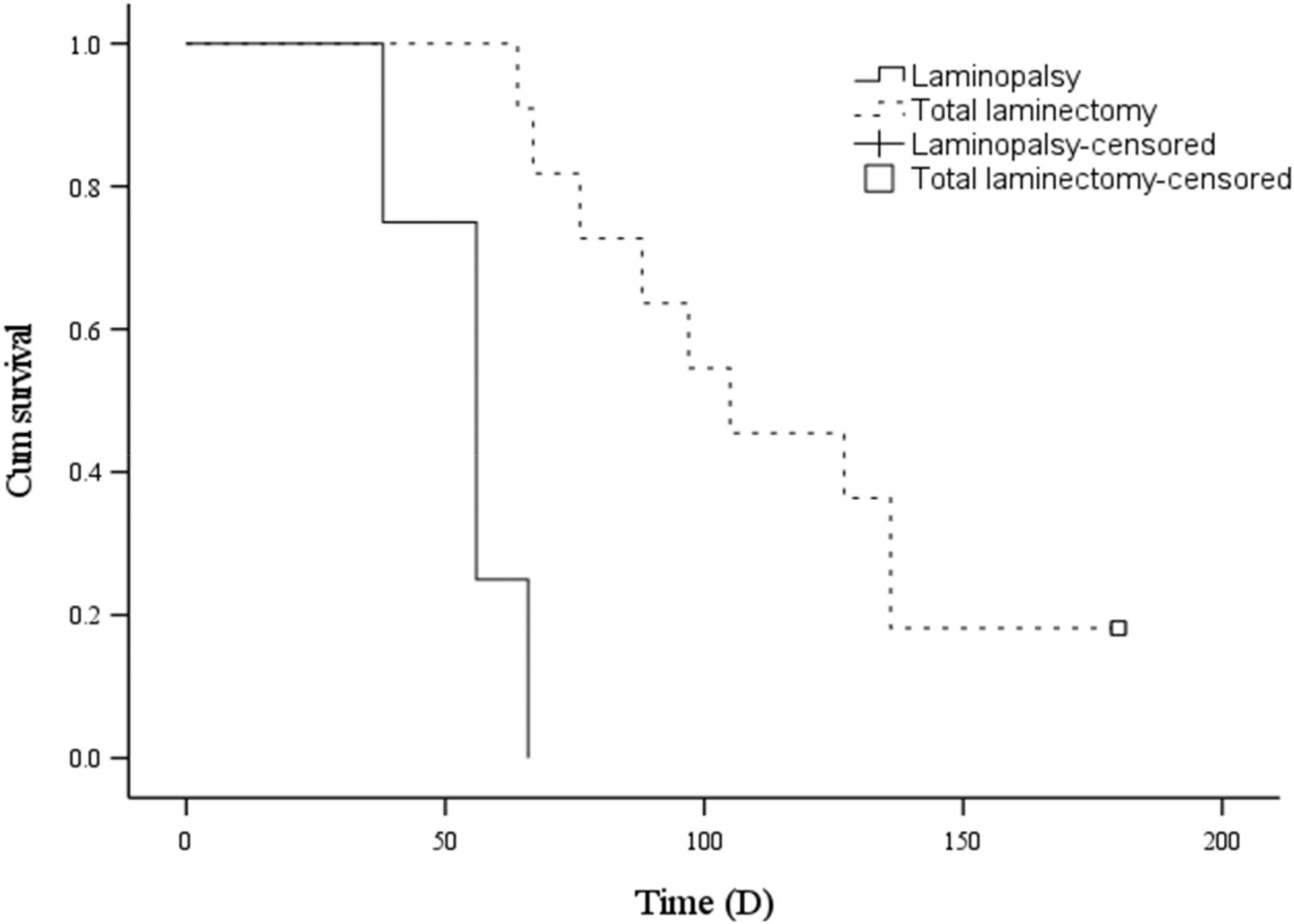
The Kaplan–Meier plot assessing the effect of surgical methods on the relief of C5P.

**Figure 6:**
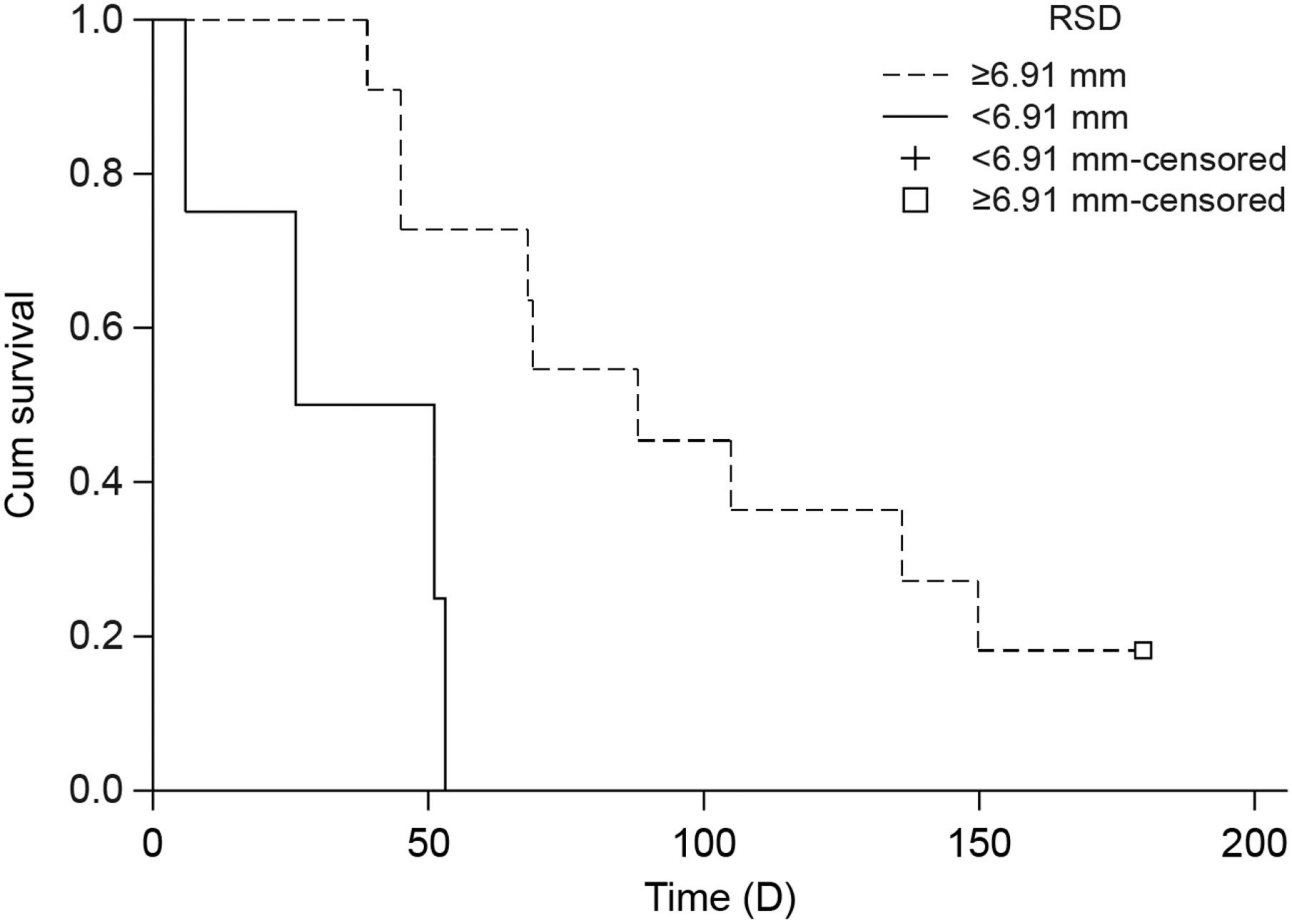
The Kaplan–Meier plot assessing the effect of the RSD on the relief of C5P.

Survival analysis was conducted on the surgical methods (Figure 5) and RSD (Figure 6). The results found that patients in laminoplasty group had a more significant improvement in C5P symptoms at 50 days post-operation. When RSD was less than 6.91 mm, the symptoms of C5P significantly alleviated at 50 days post-operation.

## 4 Discussion

Currently, posterior cervical decompression surgery is widely recognized both domestically and internationally for the treatment of CSM. Its effectiveness in achieving adequate decompression was satisfactory. However, the series of problems caused by C5P is postoperatively regrettable and remain a challenging complication for surgeons. The posterior research showed that the incidence of C5P ranges from 1.4% to 23% (10, 11). Although spontaneous relief would be observed in most patients, the course needed to take a relatively long time, ranging from 3 to 6 months (12, 13). In severe cases, symptoms may persist for more than three years. In the present study, 15 of the 97 subjects developed C5P, with an incidence of 15.46%, and the average recovery time was 67 days. This condition might easily lead surgeons to make incorrect judgments about the progression of the surgery, and patients might also be dissatisfied with surgical outcomes. Researchers have proposed many hypotheses about the pathogenesis, such as iatrogenic nerve root injury, posterior spinal cord float back, spinal cord ischemia or reperfusion injury, asymmetric drift of the spinal cord, and pre-existing foraminal stenosis (2, 10, 14–16). However, the specific pathogenesis has not yet been determined (17). Therefore, our retrospective study aimed to investigate risk factors and the pathogenesis of C5P. Our results demonstrated that larger RSD was associated with higher odds of C5P, while smaller IFD was associated with lower odds of C5P. These findings indicate that any factor inducing traction on the C5 nerve root might play a role in the development of C5P.

To our knowledge, this is the first study to report and find that patients in C5P group had higher RSD (*P* < 0.01), and parsimonious binary logistic regression analysis further showed that the RSD might be associated with increased odds of C5P (OR > 1). In the ROC analysis, when the RSD was higher than 6.91 mm, the accuracy of predicting the occurrence of C5P was 85.00%. In addition, survival analysis suggested that the recovery speed of radiculopathy symptoms in patients with an RSD > 6.91 mm was much slower than that in patients with an RSD less than 6.91 mm. Previous studies showed that spinal cord float back (3, 18–20) was the risk factor of C5P, because the fifth cervical vertebra was located at the apex of cervical lordosis, and owing to the relatively short length of the C5 nerve root, the posterior divergence of the C5 nerve root is more horizontal (10, 19, 21). These features may give rise to a tethering effect that elongates and compresses the C5 nerve root within the intervertebral foramen, consequently leading to C5P. Meanwhile, Wang et al. (6) found that the increse in CCD after posterior cervical surgery was also a risk factor for C5P. Because the cervical curvature increases postoperatively (2, 7–9), leading to the bowstring effect, there is a subsequent increase in spinal cord float back. Unlike previous studies, our study considered the fact that the change of CCD and spinal cord float back have the same effect on traction of C5 nerve root. Therefore, our findings suggested that spinal cord float back and changes in cervical curvature jointly contribute to the development of C5P via nerve root traction. Our results indicated that an RSD of 6.91 mm might be relevant to nerve root stretching. However, we acknowledge that this specific cutoff value was mathematically optimized based on our sample. In clinical practice, we suggest considering an RSD of approximately 6.91 mm as a practical “warning threshold”. When the calculated preoperative RSD exceeds this cutoff, surgeons should be highly vigilant about the risk of C5P and communicate actively with patients to reduce conflicts.

Our results found that patients in laminoplasty group had lower incidence of C5P (*P* < 0.05) and lower VAS score (*P* < 0.01). The survival analysis showed that the recovery speed of radiculopathy symptoms in laminoplasty group was faster than laminectomy group. Our findings were consistent with a previous study (22), Lin et al. (23) found that laminoplasty appeared to allow for a lower overall complication and C5 palsy rates, shorter operation time and lower blood loss. He et al. (24) reported laminectomy had longer operative durations and higher complication rates. Compared with laminoplasty, in our opinion, total laminectomy needed to remove more bony structure behind the spinal cord, resulting in a greater distance of spinal cord float back (25). Meanwhile, patients with CSM often present with cervical lordosis, and the use of screw-rod internal fixation during surgery typically helps restore the abnormal curvature to near-normal alignment (26). These factors mentioned above might lead to a greater change in the cervical curvature and result in a greater SD. Conversely, in laminoplasty, the volume of the spinal canal is expanded without artificial intervention on the cervical curvature, which relies solely on the traction of muscular soft tissue for recovery. This results in lower change in the cervical curvature and relatively less distance of posterior spinal cord float back. Therefore, we suggested that laminoplasty might have less odds of C5P compared with laminectomy.

Our results also found that patients in C5P group had less IFD (*P* < 0.01), and parsimonious binary logistic regression analysis further showed that the IFD was associated with decreased odds of C5P (OR < 1). In the ROC analysis, the cutoff value of the IFD was 2.82 mm. Our results were consistent with previous studies. IFD (27–29) was found to be a risk factor for C5P. To the best of our knowledge, no studies have cited a mechanism explanation of how C4/5 foraminal stenosis influences the occurrence of C5P. Although Lee et al. (14) suggested that C4/5 foraminal stenosis might be related to ischemic/reperfusion injury of the C5 root nerve after laminoplasty, only a small number of cases underwent foraminotomy in their study, therefore, the mechanism of how C4/5 foraminal stenosis influences the occurrence of C5P still remains unclear.

For the prevention and treatment of radiculopathy, some scholars pointed out that intraoperative electrophysiological monitoring can be used to monitor intraoperative nerve injury and prevent the occurrence of C5P (14, 30). Studies have shown that giving corticosteroids, analgesics, mecobalamin and other drugs can significantly relieve the clinical symptoms of patients with C5P (30). Physical therapy is also effective, including postoperative shoulder swing or Codman exercises (flexion, extension, and circumference), elbow flexion at the same distance, active resistance exercise, and other functional training, which can significantly reduce symptoms after 1 month (13). Prophylactic foraminoplasty (26, 31) might adopt the method of simple gun clamp gnawing decompression, which can not only fully decompress the nerve root but also preserve part of the bone volume of the spinal canal.

## 5 Limitations

Of course, there are still limitations in our study. First, the sample size was not sufficiently large (*n* = 97), and the number of positive C5P cases was limited, which may affect the statistical power of the multivariate analysis. Second, as a retrospective cohort study, the assignment of surgical methods was not randomized. Although we applied strict surgical indications, patients in the laminectomy group often presented with more complex baseline pathologies (e.g., instability or kyphosis) compared to the laminoplasty group. This inherent selection bias implies that the higher incidence of C5P in the laminectomy group might be explained by baseline disease severity, not solely the surgical technique itself. In addition, the follow-up time was insufficient and relatively short, with long intervals between follow-ups, which can lead to selection bias and subjective errors in the results. There is a lack of long-term recovery status in patients with C5P who have not recovered within 6 months after surgery, as well as a lack of follow-up status of patients over a longer period. Although we have suggested that the RSD is related to C5P, we have not yet made horizontal comparisons with other measurement methods; therefore, the reliability value still needs to be further verified. The RSD was not able to completely represent the cause of nerve root traction, while the nerve root tethering or kinking mechanisms cannot be demonstrated by direct imaging evidence, which will be an area that needs to be further improved.

## 6 Conclusion

RSD might be associated with increased odds of C5P, and the IFD might be associated with decreased odds of C5P. These results indicated that any potential factor that may induce traction on the C5 nerve root could contribute to the development of C5P. Laminoplasty might have lower odds of C5P compared with laminectomy.

## Data Availability

The original contributions presented in the study are included in the article/Supplementary Material, further inquiries can be directed to the corresponding authors.
